# Standards and quality of care for older persons in long term care facilities: a scoping review

**DOI:** 10.1186/s12877-022-02892-0

**Published:** 2022-03-19

**Authors:** Letasha Kalideen, Pragashnie Govender, Jacqueline Marina van Wyk

**Affiliations:** School of Clinical Medicine, College of Health Sciences, University of KwaZulu-Natal, Private Bag X54001, Durban, 4000 South Africa

**Keywords:** Standards of care, Long-term care facilities, Older people, Quality of care, Healthy ageing

## Abstract

**Background:**

Caring for older persons has become a global necessity to ensure functional ability and healthy ageing. It is of paramount importance that standards of care are monitored, especially for older persons who live in long term care facilities (LTCF). We, therefore, scoped and summarised evidence relating to standards and the quality of care for older persons in LTCFs in gerontological literature globally.

**Methods:**

We conducted a scoping review using Askey and O’Malley’s framework, including Levac et al. recommendations. PubMed, CINAHL, Health Sources, Scopus, Cochrane Library, and Google Scholar were searched with no date limitation up to May 2020 using keywords, Boolean terms, and medical subject headings. We also consulted the World Health Organization website and the reference list of included articles for evidence sources. This review also included peer-reviewed publications and grey literature in English that focused on standards and quality of care for older residents in LTCFs. Two reviewers independently screened the title, abstract, and full-text of evidence sources screening stages and performed the data extraction. Thematic content analysis was used, and a summary of the findings are reported narratively.

**Results:**

Sixteen evidence sources published from 1989 to 2017 met this study’s eligibility criteria out of 73,845 citations obtained from the broader search. The majority of the studies were conducted in the USA 56% (9/16), and others were from Canada, Hong Kong, Ireland, Norway, Israel, Japan, and France. The included studies presented evidence on the effectiveness of prompted voiding intervention for urinary incontinence in LTCFs (37.5%), the efficacy of professional support to LTCF staff (18.8%), and the prevention-effectiveness of a pressure ulcer programme in LTCFs (6.3%). Others presented evidence on regulation and quality of care (12.5%); nursing documentation and quality of care (6.3%); medical, nursing, and psychosocial standards on the quality of care (6.3%); medication safety using the Beer criteria (6.3%); and the quality of morning care provision (6.3%).

**Conclusion:**

This study suggests most studies relating to standards and quality of care in LTCFs focus on effectiveness of interventions, few on people-centredness and safety, and are mainly conducted in European countries and the United States of America. Future studies on people-centerdness, safety, and geographical settings with limited or no evidence are recommended.

**Supplementary Information:**

The online version contains supplementary material available at 10.1186/s12877-022-02892-0.

## Background

International demographic trends explicitly indicate that the world’s population is ageing. Estimated at 900 million in 2015, the proportion of persons aged 60 years and above is projected to rise to about 2.1 billion in the next 15 years [1]. This surge in ageing will potentially increase the demand for long term care due to a deterioration in functional capacity experienced by older persons [2]. Long term care encompasses a diversity of services, including rehabilitative, restorative and ongoing-nursing care to address individualised health, social or personal care of the aged [2]. The services are planned to help the individuals live independently and safely while performing their daily activities, which would have been difficult/impossible if they had lived alone [2, 3]. Long term care can be rendered in formal or informal settings by a variety of trained or untrained caregivers, including family members, depending on the setting.

Formal long term care facilities (LTCFs) for the aged such as nursing homes and residential care homes, supplement family members’ support for their ageing relatives by providing diverse professional services [2]. These formal LTCFs offer tailored services to their residents to meet their changing needs and that of their family members. The services in response to the need of the resident are often administered by diverse trained staff attached in the LTCFs to ensure that older people who are with or at risk of a significant or ongoing loss of intrinsic capacity can maintain a level of functional ability as stipulated by the World Health Organization (WHO) [4]. Functional disability is the prime reason for using LTCFs [2, 3]. To this end, it is of the utmost importance to assess the quality of care delivered to the aged living in LTCF by monitoring their safety, efficiency and effectiveness of practices, and people-centeredness with regard to timeliness and fairness of interventions [5]. However, to enable the delivery of quality care to residents in LTCFs, it is essential to adhere to standards and ensure that the caregiving professionals are adequately trained to adhere to set criteria.

Standards for clinical and non-clinical care are critical to maintaining healthy ageing for residents in LTCFs. A scoping review focusing on standards and the quality of care delivered to older people as residents in LTCFs is needed to synthesize and highlight gaps in the literature to facilitate or direct future research to ensure healthy ageing in line with the WHO in its global strategy and action plan [6], and the sustainable development goals Plan of Action for older persons [7]. Although several prior scoping reviews have been conducted [8–17], none of these previous reviews focused on standards and the quality of care for older residents in LTCFs. Therefore, this study aimed to scope and summarise the evidence relating to standards and the quality of care for older persons in LTCFs in the English gerontological literature worldwide.

## Methods

### Scope of review

This study adopted Arksey and O’Malley’s framework, including Levac et al. recommendations as a guide [18, 19]. This study used five of the six steps outlined in the framework as follows: identifying the research question; identifying relevant evidence sources; selecting evidence sources; charting the data, and collating, summarizing, and reporting the results [18]. This study’s protocol was developed a priori and published [20]. This study population included individuals aged 65 years or more resident in LTCFs, the concept included standards (a duty determined by a given set of circumstances that present in a particular patient, with a specific condition, at a definite time and place) for care of older persons, and the context was quality of care as per the WHO definition (the extent to which health services for individuals and populations increase the likelihood of desired health outcomes. That is, safety, effectiveness, and people-centerdness through timely, efficient, integrated, and equitable health care) [5]. Evidence sources published globally and grey literature relating to standards and quality of care of older persons in the LTCFs were included [20]. Limits included only English language publications and primary study designs [20].

### Identifying the research question

This study sought to answer the main research question: To date, what evidence and knowledge gaps exist relating to standards and the quality of care for older persons in LTCFs? The population, concept, and context framework was used to define the eligibility of this review question.

### Identifying relevant studies/evidence

We systematically searched the literature to retrieve grey literature and published studies relating to standards and the quality of care for older persons in LTCFs. We used a combination of keywords (“older person”, “aged”, “elderly”, “aging”, “ageing”, geriatric, standard of care”, “standard”, “care”, “clinical practice guideline”, “quality of care”, “long term care facility”, “long term care setting”, “nursing home”), Boolean terms (AND/OR), and Medical Subject Headings (MeSH) terms during the search [20]. This study limited the search on PubMed, EBSCOhost (CINAHL with full text and Health Sources), Scopus, Cochrane Library, and Google Scholar for relevant peer-reviewed publications in English from inception to May 2020. We also consulted the WHO website and the reference list of included articles. Each search was adequately documented (Supplementary file 1). The Peer Review of Electronic Search Strategies statement [21] guided this study’s electronic search strategy. EndNote X9 reference manager was used to compile all relevant sources of evidence and identify and remove duplicates.

### Screening and selection of studies

LK conducted the database searches and titles screening assisted by JvW after the search strategy and screening methods were piloted to calibrate operators, increase consistency, and fine-tune the methods. PG reviewed the retrieved titles to ensure completeness prior to abstract screening. Subsequently, the cleaned EndNote library was shared among the review team following the removal of duplicate titles. Using an electronic screening tool developed in Google forms, LK and JvW independently screened the abstracts, and full texts and categorized them into an “include” or “exclude” category based on the study’s eligibility criteria (scope of review). The review team resolved all discrepancies (relating to the eligibility of an evidence source) between LK and JvW at the abstract screening stage through discussions until consensus was reached, whilst PG resolved the discrepancies between LK and JvW at the full-text screening phase. Cohen’s kappa coefficient (κ) statistic was calculated to determine the inter-rater agreement between the reviewers at the full-text screening phase and Kappa statistic less than 50, 50–70%, and greater than 70% were respectively interpreted as poor, moderate, and substantial agreement. We adapted the PRISMA flow diagram to present the screening results [22].

### Charting the data

LK and DK independently extracted all relevant data from the evidence sources using a form developed in Microsoft Excel. Prior to the data extraction, the form was piloted by LK and DK using 10 % of the evidence sources to ensure accuracy and reliability of the data. LK thoroughly read the full texts and extracted all relevant data from the included studies. Inductive and deductive approaches were employed to extract relevant data. We extracted data that described the characteristics of the study: author(s) and publication year, methodological details, standards/interventions, standard procedure, and the study findings relating to the quality of care.

### Quality appraisal

We employed the mixed method quality assessment tool to conduct a quality appraisal of each included primary study [23]. Methodological quality appraisal of individual studies is not required for a scoping review study. Still, we considered it essential for inclusion to enable this study to assess the validity of conclusions drawn by each included study. LK and JvW independently conducted the methodological quality assessment and scored each included study using the screening questions and the set of quality appraisal questions prescribed by the MMAT for the study design employed (randomized controlled trial, non-randomized study, and quantitative descriptive studies). Then, an overall quality score was calculated for each retained study using the MMAT. The quality score was generated into a percentage and graded as low (less than 50%), average (50 to 75%) and high (greater than 75% as published in previous study [24].

### Collating, Summarising, and reporting the results

A content analysis [25] of the extracted studies was performed to categorized the reported standards of care into themes. A further content analysis of the findings reported for each theme was performed to link each theme to the quality component been addressed based on the WHO definition quality of care (safety, effectiveness, and people-centrerdness through timely, efficient, integrated, and equitable health care). A narrative summary of the findings for each standard (quality component been addressed) was reported. The characteristic of the included articles was described using frequencies and percentages.

## Results

**Fig. 1:**
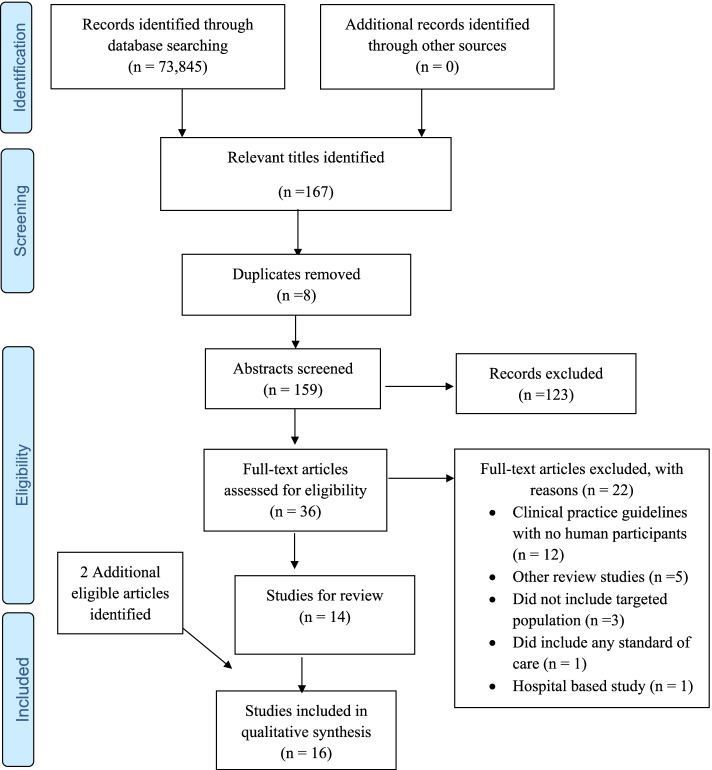
PRISMA flow diagram [21]

The broader electronic search yielded 73,845 citations, of which 167 potentially eligible titles were identified with eight duplicates. Subsequently, 123 and 22 evidence sources were removed at the abstract and full-text screening stages, respectively. Finally, 16 evidence sources, including two articles obtained from reference list searches met the inclusion criteria and were included for data extraction and review. There was substantial agreement between the reviewer’s responses at the full article screening stage (Kappa statistic = 0.85, *p* < 0.01). Twelve were clinical practice guidelines with no human participants [26–36]. Five of the evidence sources excluded at the full-text stage were other review studies [37–41], three did not include this study’s population [42–44], one was a hospital-based study [45], and one did not have any standard of care [46] (Fig. 1).

### Characteristics of the evidence sources

Of the 16 included primary studies relating standards and quality of care, approximately 56% (9) were conducted in the United States of America (USA) [47–55]. The remainder involved LTCFs in Canada [56], Hong Kong [57], Ireland [58], Norway [59], Israel [61], Japan [61], and France [62]. The majority 31% (5) of the included studies were quasi-experimental studies [47–49, 51, 53], whilst the minority (< 1%) used a qualitative study design [58]. Six of the included studies reported evidence on prompted voiding standards [47, 50–53, 57]. Other standards of care reported in the included studies were regulatory [56, 59], nursing documentation and person-centered care [58], medication safety (Beers criteria) [61], provision of professional support to LTCFs [48, 49, 62], among others, as shown in Table 1.

**Table 1:** Characteristics of the included primary studies

Author and publication year	Country	Study design	Number of study participants	Intervention/standard	Standard procedure
Bravo et al., 2002 [56]	Canada	Longitudinal study cohort	299 from 88 LTCFs	Regulation	Regulation of LTCFs
Broderick, 2013 [58]	Ireland	Qualitative study	65 records of 6 residents	Nursing documentation and person-centred care	Documentation of nursing care
Burgio et al., 1994 [47]	USA	Multiphase study (Quasi-experimental study)	41 from 1 LTCF	Prompted voiding	Treatment of urinary incontinence using different (1-h, 2-h, and 3-h) prompted voiding schedules compared to the standard 2-h schedule
Fleishman et al., 1990 [60]	Israel	Cross-sectional study	136 residents in 9 units (4 private and 5 public LTCFs)	Quality of care assessment (Tracer method)	Medical, nursing, and psychosocial standards of care (using hypertension, vision difficulties, Hearing difficulties, oral health problems, mobility problems, difficulty in washing, difficulty in dressing, difficulty in brushing teeth, urinary incontinence, feeling of loneliness, and lack of autonomy tracers)
Kirkevold and Engedal, 2006 [59]	Norway	Cross-sectional study	1926 residents	Regulation of quality of care	Patients’ right to decide when to go to bed, when to eat, when to have visitors and how to have a private life in the institution; patient’s right to have skilled help to manage dressing, personal hygiene, visits to the toilet and in preparing a meal; and patient’s right to take part in leisure activities, including going for a walk outside the institution.
Krichbaum et al., 2005 [48]	USA	Quasi-experimental study	319 residents from 3 Nursing Homes	Nursing intervention model	Two-tiered nursing intervention model of nursing care. First tier: Called for gerontological advanced practice nurses (GAPNs) to provide direct care and to teach staff to implement care protocols for residents with incontinence, pressure ulcers, depression, and aggression Second tier: Use of GAPN, the use of protocols to guide care for residents, and the addition of specific organisation-level interventions by the GAPNs.
Lai et al., 2017 [57]	Hong Kong	Randomised control trial	52 from 5 Nursing Homes	Prompted voiding interventions	Use of prompted voiding by nursing home staff in managing urinary incontinence among residents
Niwata et.al., 2006 [61]	Japan	Retrospective cross-sectional study	1669 from LTCFs	Medication safety	2003 Beers criteria for determining inappropriate medication)
Rolland et al., 2016 [62]	France	Prospective cohort study	6275 residents, 6275 from 175 Nursing Homes	Provision of Professional support to nursing home staff	Use of Global intervention comprising of professional support and education for nursing home staff on quality indicators and functional decline and emergency department transfers of residents
Ryden et al., 2000 [49]	USA	Quasi-experimental study	2 LTCF, 86 residents	Provision of Professional support to LTCFs staff (Use of Advance Practice Nurses in LTCFs)	Advanced practice gerontological nurses working with staff to implement scientifically-based protocols for incontinence, pressure ulcers, depression, and aggressive behaviour
Schnelle et al., 1989 [50]	USA	Randomised control trial	126 residents from 6 Nursing Homes	Prompted voiding treatment of urinary incontinence	Check incontinent patients on an hourly basis, ask if they needed toileting assistance (prompted), and socially reinforced for appropriate toileting
Schnelle et al., 1990 [51]	USA	Controlled experimental evaluation	126 residents from 6 Nursing Homes	Prompted voiding programme	Ask the patients on a regular schedule if they need toileting assistance
Schnelle et al., 1991 [52]	USA	Statistical quality-control	81from 4 Nursing Homes	Prompted voiding toileting procedure	Treatment of incontinence patients with prompted-voiding toileting procedure (Setting job standards by specifying how dry the patients should be if toileted on a 2-h schedule; and use of a job-monitoring control chart to continuously assess how well the job standards were being met)
Schnelle et al., 1993 [53]	USA	Quasi-experimental study	344 residents from 7 Nursing Homes	Prompted voiding programme	Prompted voiding assessment, and adherence to a 2-h prompted voiding schedule between 7:00 a.m. and 7:00 p.m.
Simmons et al., 2013 [55]	USA	Cross-sectional study	169 in 4 community LTCFs	Morning care provision	Staff assistance with either transfer out of bed, dressing, and/or incontinence
Shannon et al. 2012 [54]	USA	Randomised, controlled prospective cohort study	133 residents in 2 Nursing Homes	Prevention of Pressure Ulcer	Pressure Ulcer Prevention Programme against the Agency for Healthcare Research and Quality guidelines and a mixture of assortment of commercial skin care products, briefs, pads, and mattresses.

### Quality of evidence

**Fig. 2:**
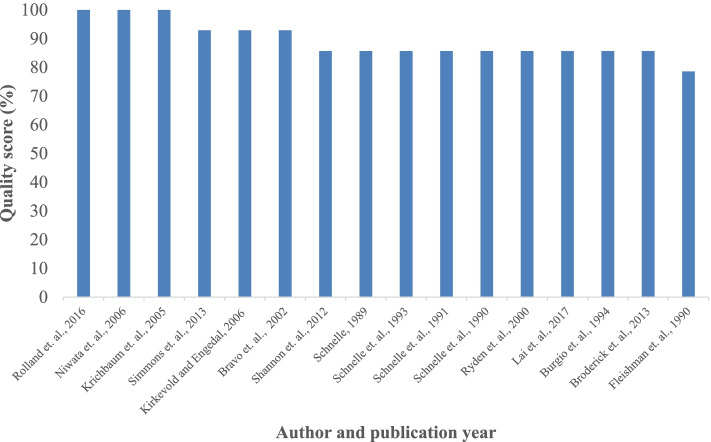
A clustered column bar chart comparing the quality score of the included studies (*n* = 16)

All 16 included studies underwent methodological quality appraisal using the MMAT (Supplementary file 2). The quality score ranged from 78.6 to 100%. Approximately 56.3% (9/16) of the included studies scored 85.7% [48, 61, 62] and 6.3% (1/16) scored 78.6% [61]. Figure 2 presents a clustered column bar chart comparing the quality scores of the included studies.

### Practice guidelines/criteria for older residents in LTCFs

Aside from the 16 included studies, this review revealed 12 practice guidelines for care of older persons in LTCFs. Namely; practice guidelines for evaluation of fever and infection [26], practice guidelines for improving medication management [27], oral health care guidelines [28, 31], and standard guidelines for specialized nutrition support [63]. Clinical practice guidelines for the evaluation of fever and infection [30], guidelines for reducing the risk of aspiration pneumonia through oral health care [31], standards for psychological services [32], infection prevention and control [33], prevention of influenza [64], and recommendations for the management of *Clostridium difficile* [34] were also revealed. The remainder was a framework to combat antimicrobial resistant bacteria [35] and criteria for determining inappropriate medication use [36]. Most of these guidelines focused on clinical care for older residents in LTCFs. This finding suggests a dearth of guidelines for non-clinical care for older people resident in LTCFs.

### Findings from the primary studies

#### Prompted voiding interventions (effectiveness)

Six of the 16 included studies highlighted evidence on prompted voiding intervention/standards [47, 50–53, 57]. Burgio et al. indicated significant improvement to the two-hourly schedule in one of the four groups involved. Two groups appeared to improve on the less intensive three-hour schedule (*P* < 0.05) [47]. Moreover, the authors indicated that during training self-initiated toileting reduced (*P* < 0.05) and volume voids in a suitable receptacle increased (*P* < 0.05) [47]. Lai et al. investigated the effectiveness of the use of prompted voiding by nursing home staff in managing urinary incontinence among residents in Hong Kong over 6 months [57]. Significant differences in wet episodes, incontinence rate, and total continent toileting per day between the control and intervention groups 6 months after intervention were noted. A reduction of 9% incontinence was noted in the intervention group [57].

Schnelle et al. appraised a prompted voiding treatment for urinary patients presenting incontinence in nursing homes in the USA by reviewing patients hourly, and ascertaining if they required toileting assistance, and socially reinforced proper toileting [50]. In their study, the frequency of incontinence per 12 h from an average of 3.85 at baseline to an average of 1.91 during the treatment [50]. Schnelle et al. reported that prompted voiding treatment significantly reduces incontinence frequency in patients who can initiate voiding when prompted in another article aimed at providing a controlled experimental evaluation of prompted voiding procedures of 126 patients [51]. They found no differences between the immediate and delayed treatment groups at baseline (Phase 1), but found significant differences in Phase 2 (F(1,125) = 33.64, *P* < 0.001) [51]. Nonetheless, the treatment effects were replicated in Phase 3 when both groups of patients received treatment with no significant differences between Phase 2 and Phase 3 (F(1,125) = 0.008, *P* < 0.931) [51]. Schnelle et al. used a statistical quality-control process to assess the effectiveness of incontinence management procedures by indigenous nursing staff in four nursing homes [52]. Their revealed that 36 out of the 81 patients were responsive to the toileting procedures [52]. The overall average expected wetness for all toileting patients was 18%, SD 16% [52]. Furthermore, Schnelle et al. reported that nearly 75% of the 344 residents significantly improved wetness, and 35% (120/344) decreased wet episodes to less than 1 per 12-h period in their study aimed to provide a specific illustration of how such management technologies can improve nursing aides’ incontinence care [53].

#### Provision of professional support to LTCF staff (effectiveness)

Three of the 16 included studies presented evidence on professional support to LTCF staff and quality of care. Rolland et al. investigated the effects of a global intervention that included professional support and education for nursing home staff on quality indicators as well as functional decline and emergency department transfers of residents [62]. At the outset, they reported that quality indicators in nursing homes in France were generally low [62]. The annual rate of transfer to the emergency department was found to be high (about 20%) in both the intervention and control groups [62]. The global intervention was found to have a significant positive effect on the prevalence of assessment of pressure ulcer risk, depression, pain, and prevalence of emergency department transfers but had no significant impact on the functional decline [62]. Ryden et al. investigated the impact on clinical outcomes when advancedpractice gerontological nurses collaborated with nursing home staff in the United States to implement evidence based protocols for incontinence, pressure ulcers, depression, and aggressive behavior [49]. Eighty-six residents who received input from gerontological advanced practice nurses (GAPNs) in their care improved significantly more (less decline in incontinence, pressure ulcers, and aggressive behavior, and higher mean composite trajectory scores); 111 compared to 111 residents who received standard care [49]. As a result, Ryden et al. proposed that GAPNs can serve as important bridges between current scientific knowledge about clinical problems and nursing home staff [49]. The effectiveness of the second tier of interventions in a two-tiered nursing intervention model designed to improve the quality of care for residents in LTCFs in the United States was tested by Krichbaum et al. [48]. The first tier of the model required GAPNs to provide direct care and teach staff how to implement care protocols for residents with incontinence, pressure ulcers, depression, and aggression, while the second tier required GAPNs to add a set of organization-level (OL) interventions such as membership on the LTCF quality assurance committee and collaboration with staff on problem solving teams [48]. In the first tier, there was a significant improvement in resident outcomes for incontinence, pressure ulcers, and aggression [48]. The addition of OL interventions also revealed a significant improvement in both depression scores and depression trajectory in LTCF residents who received OL interventions [48].

#### Effect of regulation of LTCFs (effectiveness)

Two reported evidence sources relating to regulation and quality of care in LTCFs were accessed from the 16 included studies. Bravo et al. compared the mortality rate in regulated and unregulated facilities and concluded that quality of care has a much stronger influence on resident outcomes in Canada than regulation [56]. The study found that a resident’s length of survival in LTCF is unaffected by the regulatory status of the facility where he or she lived at the start [56]. Nonetheless, residents with low quality ratings at the outset had shorter survival times than those who received good care [56]. The median survival time for residents receivinginadequate care was 28 months, compared to 41 months for those receiving adequate care (*p* = 0.0217) [56]. The Kirkevold and Engedal study described the extent to which nursing homes provided services in accordance with the ‘Regulation of quality of care’ and reported that the majority of residents in Norwegian nursing homes received good basic care [59]. However, the study found that residents had fewer opportunities to participate in leisure activities such as going for a walk [59]. Low function in mental capacity, low function in activities of daily living, and aggressive behavior in residents were found to have a strongly negative association with acceptable quality of care [59].

#### Documentation of nursing care (people-centeredness)

One of the 16 included studies demonstrated nursing documentation and quality of care. Broderick et al. investigated nursing care documentation in Ireland’s long-term care facilities and described aspects of personcentered care as evidenced in nursing records [58]. In their study, they revealed that many nursing records were incomplete and contained infrequent information about psychosocial aspects of care [58]. The nurses interacted with the residents and worked with their beliefs and values, but nursing documentation was not completed in consultation with the patient, and there was little evidence that patients were involved in care decisions [58].

#### Medical, nursing, and psychosocial standards of care (people-centeredness)

One of the 16 included studies also demonstrated findings on medical, nursing, and psychosocial standards of care quality. Fleishman et al. assessed the quality of care in Israeli LTCFs, focusing on medical, nursing, and psychosocial standards of care (using tracers such as hypertension, vision difficulties, hearing difficulties, oral health problems, mobility problems, difficulty in washing, difficulty in dressing, difficulty in brushing teeth, urinary incontinence, loneliness, and lack of autonomy) [61]. According to the Fleishman et al. study, residents in good units were more satisfied than residents in poor units [61]. Residents in independent and frail units, on the other hand, were more satisfied than residents in nursing units [61]. Loneliness, autonomy satisfaction, staff attitudes satisfaction, and living conditions were all significant predictors of overall satisfaction (R2 = 0.478, p0.001) [61].

#### Medication safety (Beers criteria) (safety)

One of the 16 included studies reported on medication safety. Niwata et.al. assessed inappropriate medication in LTCFs in Japan based on the Beers criteria. The study indicated that 356 (21.3%) of the 1669 patients were treated with potentially inappropriate medication independent of disease or condition [61]. Ticlopidine was reported as the most (107 patients (6.3%)) commonly inappropriately prescribed medication [61]. The study further indicated that 300 (18.0%) patients were treated with at least one inappropriate medication dependent on the disease or condition [61]. Factors such as psychotropic drug use (OR = 1.511), medication cost per day (OR = 1.173), number of medications (OR = 1.140), and age (OR = 0.981) related to inappropriate medication use were independent of disease or condition [61].

#### Provision morning care (staff assistance with either transfer out of bed, dressing, and/or incontinence care) (people-centeredness)

One of the 16 included studies reported on morning care. Simmons et al. examined three aspects of morning care (staff assistance with either transfer out of bed, dressing, and/or incontinence) and reported that 40% of the observations showed a lack of morning care provision, including any staff-resident communication about care, during the 4 h observation period [55]. The findings of that study reported that residents who were physically more dependent and required two members of staff for transfer were more likely not to receive morning care [55].

#### Prevention of pressure ulcer (effectiveness)

Shannon et al. 2012 [54] assessed the comparative prevention-effectiveness of a pressure ulcer prevention programme (PUPP) against the standard practice of prevention using Agency for Health Care Policy and Research guidelines, and an assortment of commercial skin care products, briefs, pads, and mattresses in the USA [54]. The study indicated that the PUPP strategy resulted in a 67% reduction in the incidence of nosocomial pressure ulcers over 6 months period for the residents [54].

## Discussion

This study scoped and summarised published evidence relating to standards and the quality of care for older persons in LTCFs in the gerontological literature globally. This review found 16 studies relating to standards and quality of care in LTCFs whichwere published between 1989 and 2017. The included studies mostly focus on effectiveness of interventions, few on people-centeredness and safety, and studies were mainly conducted in European countries and United States of America. The majority (37.5%) of the included literature demonstrated the effectiveness of prompted voiding intervention for urinary incontinence in LTCFs, provision of professional support to LTCF staff, and PUPP strategy. Within the LTCF context, this study also revealed literature on regulation and quality of care; nursing documentation and quality of care; medical, nursing, and psychosocial standards relating to the quality of care; inappropriate medication using the Beer criteria, and the quality and provision of morning care in LTCFs.

This scoping study to the best of our knowledge is the first comprehensive review of standards and quality of care for older residents in LTCFs. Nevertheless, our study findings are consistent with a previous review study on financing and regulation of oral care in LTCFs that noted that the majority of studies originated from the USA. In this study (56%) of the publications were conducted between 1989 and 2017 in the USA. Similarly, MacEntee et al., also reported that 28 of the 68 references included in their review were from the USA [8]. The included references that focused on the use of prompted voiding interventions for incontinence in older residents in LTCFs evidence their effectiveness though limited. This finding corroborates Roe et al. report that evidence on the effectiveness of voiding programmes are limited [65].

Our study finding has implications for practice and research. For instance, prompted voiding intervention for urinary incontinence, provision of professional support to LTCF staff, and PUPP strategy in LTCFs were shown to be effective by the studies included. Hence, the implementation or scale up of these interventions for older people resident in LTCFs will be useful towards maintaining healthy ageing in keeping with international goals. The adoption and implementation of these interventions in all LTCFs on a global scale would be beneficial though potential contextual challenges may need prioritizing. This study findings also showed infrequent documentation of nursing care. This is worrying since documentation of care is essential for subsequent assessment and care planning. Besides, documentation of care also helps evaluate and monitor the quality and standards of care to ensure possible improvement where needed Moreover, records of care are useful when a legal case arises against the LTCF. This study further revealed a dearth of research on psychosocial standards of care. Anxiety, depression, delirium, dementia, personality disorders, and substance abuse are common psychological issues that often affect older residents [66, 67]. Social and emotional issues may lead to loss of autonomy, grief, fear, loneliness, financial constraints, and lack of social networks [66, 67]. Therefore, standards or guidance on psychosocial care for residents in LTCFs are critical and should be considered in future research.

Moreover, this study suggests limited primary research focusing on standards and quality of care for older residents in LTCFs. Most (9/16) of the included studies were from a single country (USA), hence this study’s findings cannot be generalized for older populations resident in other countries due to differences across health systems and socioeconomic variations. Therefore, future international studies are needed involving standards and care for older persons using varying study designs to provide contextualized evidence relating to the quality of care. This review also suggests a dearth of research on standards and quality of care for older residents especially in low to middle-income countries (LMICs).. This is a concern considering that the population of older persons in LMICs is said to be on a rise [1]. Besides the nature of work and migration of people, the traditional extended family system is no longer a dominant social structure, making older persons vulnerable in LMICs. Hence, older people may have to relocate to LTCFs to facilitate healthy ageing due to inadequate or lack of home-based care. To this end, several primary studies are needed to provide evidence on the standards and quality of care of older persons in LMICs, and the lived experiences of older residents living in LTCF in LMICs. The evidence emanating from such future studies will help improve the quality of care delivered to older residents in LTCFs in LMICs. Research on areas such as oral and nutritional standard of care is needed since no study on these areas met this study’s inclusion criteria. The research, alongside political will and commitment to improving the quality of care for older persons in LTCFs are essential to enable healthy ageing in line with the WHO global strategy and action plan [6] and the SDG Action Plan for older persons [7].

This scoping review study has many strengths. It is potentially the first exhaustive review to focus on standards and quality of care for older residents in LTCFs. This study has demonstrated the available evidence in the literature and knowledge gaps. This study included literature worldwide. This scoping review followed most of the steps required of a systematic review, including the methodological appraisal of the included references. Despite these strengths, our review has many limitations. Only a few databases were searched. It is possible that other useful articles relating to standards and quality existed in those databases not included in this study. Perhaps, our study eligibility criteria such as limitations to only English language publications, also excluded useful evidence published in other languages published elsewhere. Moreover, we included only primary studies which resulted in the exclusion of many other review studies and guideline documents. Notwithstanding these limitations, this scoping review has synthesized the knowledge from the existing literature relating to the care for older residents in LTCFs. It has also provided useful evidence to guide future research.

## Conclusion

This study synthesized evidence on useful standards and highlighted gaps in the literature on quality of care. However, the findings suggest Tthat most studies relating to standards and quality of care in LTCFs focus on the effectiveness of interventions, few on people-centeredness and safety, and mainly conducted in European countries and United States of America. Future studies focusing on people-centeredness, safety, and geographical settings with limited or no evidence are recommended. Research using various primary study designs are needed to inform the standards and quality of care for older people resident in LTCFs, particularly in LMICs.

## Supplementary Information

**Additional file 1.**

**Additional file 2.**

## Data Availability

We have duly cited all articles and data is presented in a form of references.

## Abbreviations

GAPN: Gerontological Advanced Practice Nurse
LTCF: Long Term Care Facility
MMAT: Mixed-Method Appraisal Tool
OL: Organization Level
PUPP: Pressure Ulcer Prevention Programme
USA: United States of America
WHO: World Health Organization
