# Supplementary material for: Standards and quality of care for older persons in long term care facilities: a scoping review
Source: BMC Geriatr. 2022 Mar 19;22:226. doi: 10.1186/s12877-022-02892-0 (PMC8933989; doi:10.1186/s12877-022-02892-0)
Supplement: Supplementary file 1 — Additional file 1. [file 12877_2022_2892_MOESM1_ESM.docx]

**Standards and Quality of Care for the Elderly in Long Term Care Facilities: A Scoping Review**

Letasha Kalideen^1^, Pragashnie Govender^1^, Jacqueline Marina van Wyk^1^

^1^School of Clinical Medicine, College of Health Sciences, University of KwaZulu-Natal, Private Bag X54001, Durban, 4000, South Africa

**Supplementary file 1:** Electronic databases search and title screening results

| **Date** | **Databases** | **Keywords** | **Search results** | **Number of eligible titles** |
| --- | --- | --- | --- | --- |
| 18/05/2020 | SCOPUS | "standard of care" OR clinical practice guidelines AND quality of care OR quality of service AND elderly OR geriatrics AND long-term care | 1,436 | 38 |
|  | Google Scholar | standard of care OR ( clinical practice guidelines or best practice or clinical practice ) AND ( quality of care or patient care or quality of service ) AND ( aging or ageing or elderly or older adults or seniors or geriatrics ) AND ( long-term care or nursing home or residential care or assisted living ) | 2, 350 | 50 |
| 21/05/2020 | PubMed | ((((((((("standard of care"[MeSH Terms] OR ("standard"[All Fields] AND "care"[All Fields]) OR "standard of care"[All Fields]) OR ("practice guideline"[All Fields] OR "practice guidelines as topic"[MeSH Terms] OR "clinical practice guideline"[All Fields])) OR (standard[All Fields] AND ("methods"[MeSH Terms] OR "methods"[All Fields] OR "procedure"[All Fields]))) OR (standard[All Fields] AND operating[All Fields] AND ("methods"[MeSH Terms] OR "methods"[All Fields] OR "procedure"[All Fields]))) AND "quality of care"[All Fields]) AND ("aged"[MeSH Terms] OR "aged"[All Fields] OR "elderly"[All Fields])) AND ("aging"[MeSH Terms] OR "aging"[All Fields] OR "ageing"[All Fields])) AND geriatric[All Fields]) AND "long term care facility"[All Fields]) OR "long term care setting"[All Fields] OR ("nursing homes"[MeSH Terms] OR ("nursing"[All Fields] AND "homes"[All Fields]) OR "nursing homes"[All Fields] OR ("nursing"[All Fields] AND "home"[All Fields]) OR "nursing home"[All Fields]) | 8,069 | 59 |
| 22/05/2020 | Ebscohost (Academic search complete, CINAHL with full text, Health Resources) | standard of care OR ( clinical practice guidelines or best practice or clinical practice ) AND ( quality of care or patient care or quality of service ) AND ( aging or ageing or elderly or older adults or seniors or geriatrics ) AND ( long-term care or nursing home or residential care or assisted living ) | 61,366 | 16 |
| 23/05/2020 | Cochrane Library | standard of care or clinical practice guidelines or best practice or clinical practice in Title Abstract Keyword AND quality of care in Keyword AND aging or ageing or elderly or older adults or seniors or geriatrics in Keyword AND long-term care or nursing home or residential care or assisted living in Title Abstract Keyword - (Word variations have been searched) | 624 | 4 |
| Total |  |  | 73,845 | 167 |
